# A retrospective analysis of the role of age and sex in outcomes of non-surgical periodontal therapy at a single academic dental center

**DOI:** 10.1038/s41598-024-60094-7

**Published:** 2024-04-25

**Authors:** Nikola Angelov, Nikolaos Soldatos, Effie Ioannidou, Tonia C. Carter, Neel Shimpi, Joseph Applegate, Krishna Kumar Kookal, Karo Parsegian

**Affiliations:** 1 Department of Periodontics and Dental Hygiene, UTHealth Houston School of Dentistry, Houston, TX USA; 2 Division of Periodontics, Department of Regenerative and Reconstructive Sciences, School of Dentistry, Oregon Health & Science University, Portland, OR USA; 3 Department of Orofacial Sciences, School of Dentistry, University of California San Francisco, San Francisco, CA USA; 4 Center for Precision Medicine Research, Marshfield Clinic Research Institute, Marshfield, WI USA; 5 Center for Dental Benefits, Coding and Quality, American Dental Association, Chicago, IL USA; 6 Biomedical Informatics Group-Analytics Research Center, UTHealth Houston School of Dentistry, Houston, TX USA; 7 Division of Periodontics, Department of Diagnostic Sciences and Surgical Dentistry, School of Dental Medicine, University of Colorado Anschutz Medical Campus, 13065 E 17th Ave, Rm 130J, Mail Stop F847, Aurora, CO 80045-2532 USA; 8 Technology Services and Informatics, UTHealth Houston School of Dentistry, Houston, USA

**Keywords:** Aging, Association, Sex, Periodontitis, Treatment outcome, Periodontitis, Dental epidemiology

## Abstract

The present study examined the role of age and sex in the outcomes of non-surgical periodontal therapy (NSPT). De-identified demographic and periodontal characteristics of patients who presented for baseline periodontal evaluation, NSPT, and periodontal re-evaluation were abstracted from electronic health records. Independent associations of age and sex with severe periodontitis defined as ≥ 5 mm clinical attachment loss (CAL) and ≥ 6 mm probing depth (PD) were determined using multinomial logistic regression. The null hypothesis was rejected at α < 0.05. A total of 2866 eligible subjects were included in the analysis. Significantly lower odds of CAL ≤ 4 mm than CAL ≥ 5 mm (*reference*) were observed in adults aged 35–64 (odds ratio, OR, 0.19; 95% confidence interval, CI 0.13, 0.29) and ≥ 65 years (OR 0.13; 95% CI 0.07, 0.25) compared to those aged 18–34 years. Odds of PD < 4 mm versus PD ≥ 6 mm (*reference*) were lower in adults aged 35–64 years than those aged 18–34 years (OR 0.71; 95% CI 0.55, 0.90) and higher in females compared to males (OR 1.67; 95% CI 1.14, 2.44). These results suggest more compromised post-NSPT outcomes in older adults and males compared to the respective populations and highlight the need for personalized therapeutic strategies in these populations.

## Introduction

Periodontitis is a lifelong inflammatory disease of tooth-supporting tissues^1,2^ that develops as a result of the interaction of periodontal pathogens in the dental biofilm at the gingival crevice and periodontal pocket site with a susceptible host^3,4^. Non-surgical periodontal therapy (NSPT) consists of scaling and root planing (deep tooth cleaning below the gingival line) to eliminate the microbial etiology (a bacterial biofilm), improve periodontal clinical parameters, and ultimately prevent tooth loss^5,6^. The response to the treatment is assessed 4–6 weeks post-treatment and is based on the improvement of clinical parameters^6^. Furthermore, the control of periodontal disease progression is sustained through periodontal maintenance visits at a three-month interval, which prevent disease relapse via mechanical removal of bacterial biofilm and calculus deposits and comprehensive oral hygiene reinforcement^6^. The outcomes of NSPT depend on various local and systemic predisposing factors, including the quality of mechanical instrumentation^7^, the rate of repopulation of root surfaces by pathogenic microbiota^8^, and the presence of various predisposing factors^9^.

Although dentate adults of all ages can be affected by periodontitis, its increased prevalence and/or severity are associated with older age (≥ 65 years) and male sex. National Health Examination Survey I (1960–1962) and National Health and Nutrition Examination Survey, NHANES, I (1971–1974) studies showed a higher prevalence of periodontitis in all-age males and older males compared with the respective female populations^10^. More recent 2009–2014 NHANES III-based data demonstrated a higher prevalence of total and severe periodontitis in all-age and older males compared with the respective female populations^11,12^. The increased severity of periodontitis in older adults was further demonstrated at the level of clinical attachment loss (CAL)^12^, probing depth (PD)^12^, alveolar bone levels^13^, the number of missing teeth^11^, and the tooth-specific incidence/progression of periodontitis^14^. Evidence for the essential role of aging and sex in the development and progression of periodontitis was also described in detail in a recent study^15^.

The increased prevalence and/or severity of periodontitis in older adults and males suggests that they represent higher-risk vulnerable categories of patients with periodontitis. It also raises a concern that the outcomes of NSPT in these populations can be compromised compared with younger adults and females, respectively. Although several studies supported that conclusion^16,17^, other studies did not find such a correlation^18,19^. These inconsistencies could be due to differences in study designs, study populations, definitions of periodontitis, data collection time points, and sample sizes. Additional layers of bias could result from the overrepresentation of periodontitis-associated risk and predisposing factors, the lack of stratification of patients with periodontitis based on the severity categories, and the inclusion of surgical periodontal therapy in treatment protocols. The present study aimed to examine the role of age and sex as independent predictors of outcomes of NSPT.

## Methods

### Study design

This is a retrospective observational study of de-identified patient data from the electronic health records (EHRs) of the UTHealth Houston School of Dentistry (Houston, TX, USA). All experimental protocols were approved by the Committee for the Protection of Human Subjects (CPHS), which serves as the Institutional Review Board (protocol #HSC-DB-18-0663), and were carried out in accordance with relevant CPHS guidelines and regulations. The need to obtain informed consent was waived from all subjects and/or their legal guardian(s) by the CPHS.

### Study population

*Inclusion criteria*: (i) the completed comprehensive periodontal evaluation (D0180 based on Current Dental Terminology, CDT) between January 1, 2007, and November 11, 2020, followed by scaling and root planing (SRP, D4341, and/or D4342 CDT) and periodontal re-evaluation (D4132 based on institutional internal code) completed 4–6 weeks after the last episode of SRP. If a patient had several SRP treatment plans throughout the data collection time frame, the most recent D0180, D4341, D4342, and D4132 codes were recorded; (ii) ≥ 18 years old at the time of D0180. Subjects aged 18–34, 35–64, and ≥ 65 years were stratified into young, middle-aged, and elderly groups, respectively; (iii) self-reported males and females; (iv) all self-reported races (African American, Asian, Hispanic, Caucasian, and Other including multiracial subjects); (v) American Society of Anesthesiologists physical status I, II, and III; and (vi) completed full-mouth periodontal chart and medical form at the time of each appointment. Some subjects had missing values for age, sex, or race (as indicated in the footnote of tables). Individuals who did not meet the inclusion criteria were excluded.

### Extracted variables

The following independent variables were extracted: (i) demographic characteristics (age, sex, race) and (ii) all self-reported systemic diseases recorded in the medical form. The following dependent variables were measured at six sites per tooth: CAL, PD, the distance from a free gingival margin to the cementoenamel junction (FGM-CEJ), and the number of missing teeth (excluding third molars). The deepest CAL or PD measurements were selected among the six sites at each tooth, and the mean CAL and PD were calculated as the average CAL and PD values, respectively, over all measured teeth for all subjects.

### Outcome ascertainment

The differences in baseline and post-NSPT (re-evaluation) CAL and PD values served as primary study outcomes.

### Null hypothesis

Age and sex do not affect the outcomes of NSPT in academic settings.

### Statistical analyses

The distributions of continuous and categorical variables were inspected using univariate and bivariate analyses. Categorical variable distributions were compared among categories of PD or CAL at baseline and re-evaluation using Pearson’s Chi-squared test. The mean values of CAL and PD were compared between baseline and re-evaluation using the paired samples *t*-test. The difference in PD or CAL between baseline and re-evaluation was calculated as the value at baseline minus the value at re-evaluation and compared between categories of variables using a one-way analysis of variance. Linear regression, performed separately for CAL and PD, was used to determine whether the difference in CAL and PD values between baseline and re-evaluation was associated with age (18–34, 35–64, and ≥ 65 years), sex (male or female), race (African American, Asian, Caucasian, Hispanic, Other), self-reported smoking (yes or no), diabetes mellitus (DM, yes or no), and dialysis (yes or no). Multinomial logistic regression was performed separately for CAL and PD at re-evaluation, and these models included the same variables as the linear regression models. For CAL, multinomial regression was used to model classification in the CAL ≤ 2 mm and CAL 3–4 mm categories at re-evaluation, with the reference category being CAL ≥ 5 mm at re-evaluation. For PD, multinomial regression was used to model classification in the PD < 4 mm, PD 4 mm, and PD 5 mm categories at re-evaluation, with the reference category being PD ≥ 6 mm at re-evaluation. All statistical tests were two-sided, and the null hypothesis was rejected at α < 0.05.

## Results

### Subject characteristics at baseline

At baseline, the study population consisted of 3,186 eligible subjects whose demographic characteristics are shown in Table 1. Among these subjects, 70.8% were aged ≥ 35 years, 57.3% were females, 65.4% were non-Caucasians, 20.9% were smokers, 15.9% reported having DM, and 4.0% reported undergoing dialysis. CAL ≥ 5 mm and PD ≥ 6 mm were observed in 80.6% and 67.5% of subjects, respectively, and the number of missing teeth was 7.41 ± 5.71 (mean ± SD). Age, sex, race, and self-report of DM were associated with CAL, and age and race with PD, at baseline (Table 2). The proportion of subjects aged 35–64 or ≥ 65 years was higher in the ≥ 5 mm CAL category and the proportion aged 35–64 years was higher in the ≥ 6 mm PD category, compared with other CAL and PD categories, respectively. The proportions of males and subjects with self-reported DM were also higher in the ≥ 5 mm CAL category, compared with the other CAL categories. The PD 5 mm and 6 mm categories had a larger percentage of African Americans than the PD 4 mm category (19.1 and 21.7%, and 15.0%, respectively), whereas the PD 4 mm and 5 mm categories had a larger percentage of Caucasians than the PD ≥ 6 mm category (41.2, 37.8, and 32.8%, respectively; *P* = 0.0328).

**Table 1:** Demographic characteristics of study subjects^a^.

Characteristics^b^	Value *n* (%)
Age (years)	
18–34	911 (29.2)
35–64	1522 (48.7)
≥ 65	692 (22.1)
Sex	
Male	1354 (42.7)
Female	1820 (57.3)
Race	
African American	590 (20.6)
Asian	241 (8.4)
Caucasian	992 (34.6)
Hispanic	957 (33.4)
Other	84 (3.0)
Baseline CAL (mm)	
1–2	52 (1.6)
3–4	567 (17.8)
≥ 5	2567 (80.6)
Baseline CAL (mm), mean ± SD	2.64 ± 1.25
Baseline PD (mm)	
4	204 (6.4)
5	832 (26.1)
≥ 6	2150 (67.5)
Baseline PD (mm), mean ± SD	3.19 ± 0.62
Missing teeth, mean ± SD	7.41 ± 5.71
Smoking	
Yes	666 (20.9)
No	2520 (79.1)
Diabetes mellitus	
Yes	507 (15.9)
No	2679 (84.1)
Dialysis	
Yes	128 (4.0)
No	3058 (96.0)

^a^The analyses included the 3,186 subjects who presented for non-surgical periodontal therapy.

^b^Number of missing values were 61, 12, and 322 for age, sex, and race, respectively.

**Table 2:** Comparison of demographic characteristics by CAL and PD categories at baseline^a^.

Characteristics^b^	CAL (mm)			*P* value^c^
	1–2 (*n* = 52)	3–4 (*n* = 567)	≥ 5 (*n* = 2,567)	
Age (years)				< 0.0001*
18–34	30 (57.7)	283 (51.1)	598 (23.7)	
35–64	13 (25.0)	205 (37.0)	1,304 (51.8)	
≥ 65	9 (17.3)	66 (11.9)	617 (24.5)	
Sex				0.0007*
Male	16 (30.8)	206 (36.5)	1,132 (44.3)	
Female	36 (69.2)	359 (63.5)	1,425 (55.7)	
Race				0.0206*
African American	11 (23.4)	112 (21.7)	467 (20.3)	
Asian	5 (10.6)	34 (6.6)	202 (8.8)	
Caucasian	15 (31.9)	150 (29.0)	827 (35.9)	
Hispanic	16 (34.1)	205 (39.6)	736 (32.0)	
Other	0 (0.0)	16 (3.1)	68 (3.0)	
Smoking				0.4448
Yes	8 (15.4)	112 (19.8)	546 (21.3)	
No	44 (84.6)	455 (80.2)	2,021 (78.7)	
Diabetes mellitus				0.0001*
Yes	3 (5.8)	62 (10.9)	442 (17.2)	
No	49 (94.2)	505 (89.1)	2,125 (82.8)	
Dialysis				0.2904
Yes	0 (0.0)	21 (3.7)	107 (4.2)	
No	52 (100.0)	546 (96.3)	2460 (95.8)	

Characteristics^d^	PD (mm)			*P* value^c^
	4 (*n* = 204)	5 (*n* = 832)	≥ 6 (*n* = 2,150)	
Age (years)				0.0085*
18–34	49 (24.4)	262 (32.2)	600 (28.4)	
35–64	91 (45.3)	374 (46.0)	1057 (50.1)	
≥ 65	61 (30.3)	177 (21.8)	454 (21.5)	
Sex				0.0522
Male	79 (38.7)	331 (39.8)	944 (44.1)	
Female	125 (61.3)	500 (60.2)	1195 (55.9)	
Race				0.0328*
African American	28 (15.0)	142 (19.1)	420 (21.7)	
Asian	17 (9.1)	60 (8.1)	164 (8.5)	
Caucasian	77 (41.2)	282 (37.8)	633 (32.8)	
Hispanic	59 (31.5)	248 (33.3)	650 (33.6)	
Other	6 (3.2)	13 (1.7)	65 (3.4)	
Smoking				0.1610
Yes	50 (24.5)	158 (19.0)	458 (21.3)	
No	154 (75.5)	674 (81.0)	1,692 (78.7)	
Diabetes mellitus				0.8764
Yes	32 (15.7)	128 (15.4)	347 (16.1)	
No	172 (84.3)	704 (84.6)	1803 (83.9)	
Dialysis				0.0979
Yes	13 (6.4)	38 (4.6)	77 (3.6)	
No	191 (93.6)	794 (95.4)	2073 (96.4)	

*P* values labeled in bold and with an asterisk (*) show statistical significance (*P* < 0.05).

^a^The analyses included the 3,186 subjects who presented for non-surgical periodontal therapy.

^b^Missing values: 5 for race at CAL 1-2 mm; 13 for age, 2 for sex, and 50 for race at CAL 3-4 mm; 48 for age, 10 for sex, and 267 for race at CAL ≥ 5 mm.

^c^Chi-squared test used to compare distributions of characteristics among categories of CAL and PD.

^d^Missing values: 3 for age and 17 for race at PD 4 mm; 19 for age, 1 for sex, and 87 for race at PD 5 mm; 39 for age, 11 for sex, and 218 for race at PD ≥ 6 mm.

### CAL and PD changes between baseline and re-evaluation

**Figure 1:**
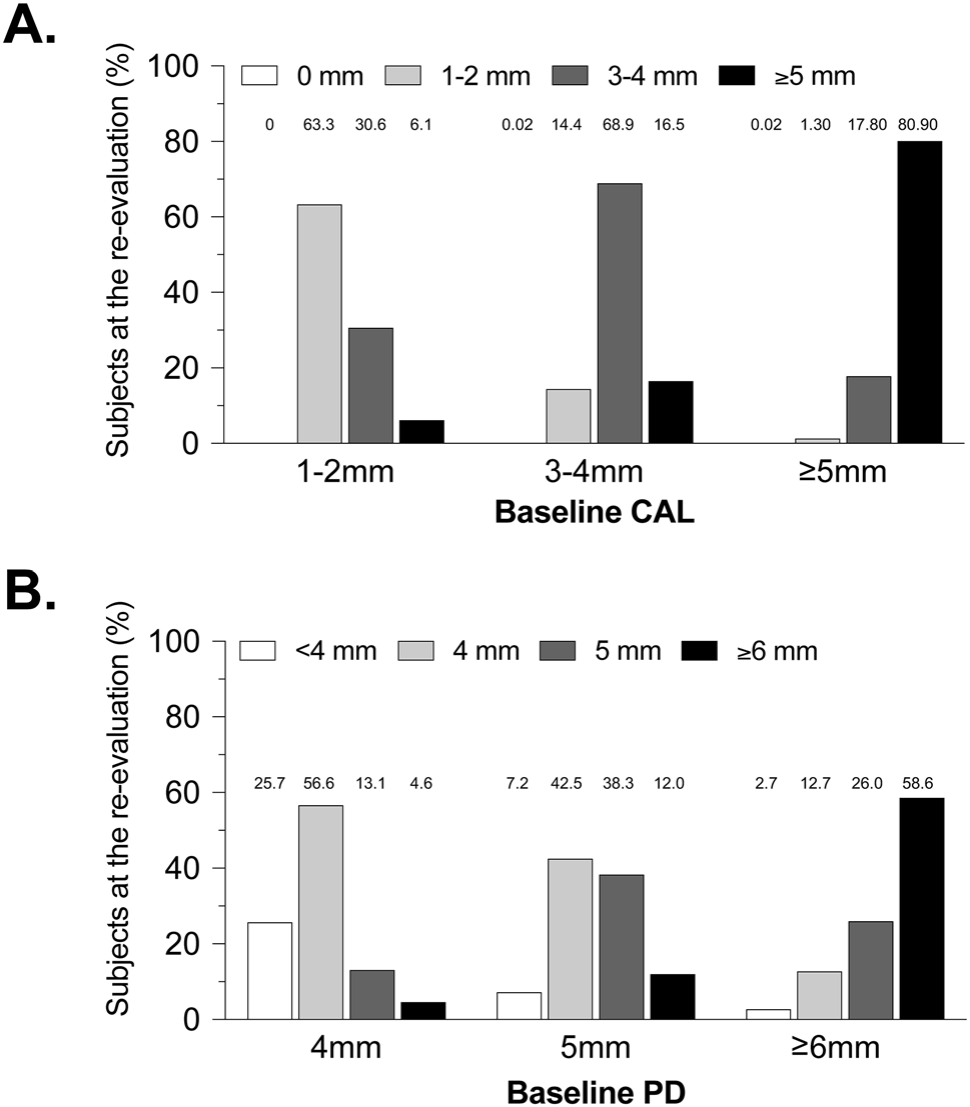
Changes in CAL and PD at re-evaluation according to categories of CAL and PD at baseline. Categories of CAL (Panel A) and PD (Panel B) at re-evaluation are shown as bars of different shades. For each CAL and PD category at baseline, the numbers above each bar represent the percentages of subjects in the respective CAL and PD category at re-evaluation. The bar heights within a baseline category total 100%. Data are for 2866 subjects who presented at baseline and re-evaluation. The numbers of subjects in baseline CAL categories were 49 for 1–2 mm, 527 for 3–4 mm, and 2,290 for ≥ 5 mm. The numbers of subjects in baseline PD categories were 175 for 4 mm, 767 for 5 mm, and 1,924 for ≥ 6 mm.

A subset of 2866 subjects presented for periodontal re-evaluation. For these subjects, mean CAL values at baseline and re-evaluation were 2.61 and 2.31 mm (*P* < 0.0001); and mean PD values were 3.18 and 2.84 mm (*P* < 0.0001), respectively (comparisons performed using a two-tailed, paired samples *t*-test). Most subjects in a CAL category at baseline were in the same CAL category at re-evaluation (Fig. 1A). For example, of the 527 subjects in the CAL 3–4 mm category at baseline, 0.2, 14.4, 68.9, and 16.5% were in the CAL 0 mm, 1–2 mm, 3–4 mm, and ≥ 5 mm categories, respectively, at re-evaluation. Subjects in a PD category at baseline tended to be in the same PD category or the adjacent, lower category at re-evaluation (Fig. 1B). For example, of the 767 subjects in the PD 5 mm category at baseline, 7.2%, 42.5, 38.3, and 12.0% were in the PD < 4 mm, 4 mm, 5 mm, and ≥ 6 mm categories, respectively, at re-evaluation.

### Factors associated with the size of CAL and PD changes between baseline and re-evaluation

No association was observed between the subject characteristics studied and the magnitude of the difference in CAL between baseline and re-evaluation in bivariate analyses (Table 3). Age, race, and smoking were associated with the magnitude of the difference in PD between baseline and re-evaluation (Table 3). The mean differences decreased with increasing age (*P* = 0.0015), and African Americans had a greater mean change compared to subjects who were Asian, Caucasian, Hispanic, or the Other race (*P* = 0.0200). Smokers also had a greater mean change than non-smokers (*P* = 0.0271).

**Table 3:** The magnitude of change (Δ) in CAL and PD between baseline and re-evaluation^a^.

Characteristics^b^	*n*	Δ CAL (mm)^c^	*P* value^d^	Δ PD (mm)^c^	*P* value^d^
Age (years)			0.5218		0.0015*
18–34	820	0.29 ± 0.67		0.37 ± 0.39	
35–64	1,369	0.31 ± 0.67		0.35 ± 0.43	
≥ 65	624	0.28 ± 0.64		0.29 ± 0.40	
Sex			0.8514		0.4078
Male	1,223	0.30 ± 0.66		0.35 ± 0.43	
Female	1,631	0.30 ± 0.67		0.33 ± 0.40	
Race			0.1233		0.0200*
African American	532	0.34 ± 0.69		0.38 ± 0.41	
Asian	217	0.36 ± 0.75		0.34 ± 0.43	
Caucasian	879	0.27 ± 0.65		0.30 ± 0.39	
Hispanic	868	0.28 ± 0.63		0.34 ± 0.42	
Other	82	0.23 ± 0.63		0.32 ± 0.41	
Smoking			0.0585		0.0271*
Yes	595	0.35 ± 0.72		0.37 ± 0.42	
No	2,271	0.29 ± 0.65		0.33 ± 0.41	
Diabetes mellitus			0.9062		0.3467
Yes	454	0.30 ± 0.71		0.36 ± 0.42	
No	2,412	0.30 ± 0.65		0.34 ± 0.41	
Dialysis			0.4088		0.2227
Yes	111	0.25 ± 0.70		0.29 ± 0.47	
No	2,755	0.30 ± 0.66		0.34 ± 0.41	

*P* values labeled in bold and with an asterisk (*) show statistical significance (*P* < 0.05).

^a^The analyses included only the 2866 subjects who had periodontal re-evaluation following non-surgical periodontal therapy.

^b^Missing values were 53 for age, 12 for sex, and 288 for race.

^c^Results represent mean ± standard deviation (SD). Difference calculated as value at baseline minus value at re-evaluation.

^d^Comparison of mean values between the categories of each variable using one-way analysis of variance.

Linear regression analysis showed that older age was independently associated with the size of the change in PD between baseline and re-evaluation (Table 4). The size of the difference decreased for subjects aged ≥ 65 years compared with those aged 18–34 years (*P* = 0.0005) when the other variables in the model were held at a constant value. Race and smoking status also showed associations in linear regression analysis. African Americans had an increased magnitude of change in PD compared with Caucasians (*P* = 0.0013), and the size of the change in CAL significantly increased for those with African American (*P* = 0.0246) or Asian (*P* = 0.0223) race (compared with Caucasians) and significantly decreased for non-smokers compared with smokers (*P* = 0.0484).

**Table 4:** Linear regression of difference between baseline and re-evaluation CAL and PD values^a^.

Characteristic	CAL		PD	
	Coefficient estimate (95% CI)	*P* value	Coefficient estimate (95% CI)	*P* value
Age (years)				
18–34	*Reference*	–	*Reference*	–
35–64	0.018 (− 0.044, 0.080)	0.5761	− 0.030 (− 0.068, 0.009)	0.1310
≥ 65	− 0.011 (− 0.089, 0.067)	0.7822	− 0.085 (− 0.134, − 0.037)	0.0005*
Sex				
Male	*Reference*	–	*Reference*	–
Female	− 0.004 (− 0.057, 0.050)	0.8973	− 0.016 (− 0.049, 0.017)	0.3315
Race				
Caucasian	*Reference*	–	*Reference*	–
African American	0.083 (0.011, 0.156)	0.0246*	0.073 (0.029, 0.118)	0.0013*
Asian	0.119 (0.017, 0.222)	0.0223*	0.045 (− 0.018, 0.108)	0.1650
Hispanic	0.024 (− 0.042, 0.091)	0.4739	0.030 (− 0.011, 0.071)	0.1454
Other	− 0.035 (− 0.188, 0.119)	0.6577	0.012 (− 0.083, 0.106)	0.8115
Smoking				
Yes	*Reference*	–	*Reference*	–
No	− 0.067 (− 0.133, − 0.0005)	0.0484*	− 0.038 (− 0.079, 0.003)	0.0708
Diabetes mellitus				
Yes	*Reference*	–	*Reference*	–
No	− 0.026 (− 0.099, 0.0463)	0.4761	− 0.045 (− 0.090, 0.000)	0.0501
Dialysis				
Yes	*Reference*	–	*Reference*	–
No	0.026 (− 0.106, 0.159)	0.6951	0.037 (− 0.045, 0.119)	0.3713

*P* values labeled in bold and with an asterisk (*) show statistical significance (*P* < 0.05).

^a^The analyses included only the 2866 subjects who had periodontal re-evaluation following non-surgical periodontal therapy.

### Factors associated with CAL and PD categories at re-evaluation

Age and sex were both associated with CAL and PD categories at re-evaluation in bivariate analyses that did not adjust for potential confounders (Table 5). The proportion of subjects who were 35–64 or ≥ 65 years of age was higher in the ≥ 5 mm CAL category and the proportion 35–64 years of age was higher in the ≥ 6 mm PD category, compared with the other CAL and PD categories, respectively. Males also formed a greater proportion of the CAL ≥ 5 mm and PD ≥ 6 mm categories, compared with the other CAL and PD categories, respectively. Smoking and a self-report of DM were also associated with being in the CAL ≥ 5 mm category, and African American race and a self-report of dialysis with being in the PD ≥ 6 mm category.

**Table 5:** Comparison of demographic characteristics by CAL and PD categories at re-evaluation^a^.

Characteristics^b^	CAL (mm)				*P* value^c^
	0 (*n* = 2)	1–2 (*n* = 136)	3–4 (*n* = 785)	≥ 5 (*n* = 1,943)	
Age (years)					< 0.0001*
18–34	1 (50.0)	81 (60.4)	330 (43.1)	408 (21.3)	
35–64	1 (50.0)	40 (29.9)	321 (41.9)	1,007 (52.7)	
≥ 65	0 (0.0)	13 (9.7)	115 (15.0)	496 (26.0)	
Sex					< 0.0001*
Male	1 (50.0)	48 (35.3)	287 (36.7)	887 (45.9)	
Female	1 (50.0)	88 (64.7)	496 (63.3)	1,046 (54.1)	
Race					0.0956
African American	0 (0.0)	28 (22.0)	149 (21.1)	355 (20.4)	
Asian	1 (50.0)	7 (5.5)	57 (8.0)	152 (8.7)	
Caucasian	0 (0.0)	36 (28.4)	229 (32.4)	614 (35.3)	
Hispanic	1 (50.0)	54 (42.5)	255 (36.1)	558 (32.0)	
Other	0 (0.0)	2 (1.6)	17 (2.4)	63 (3.6)	
Smoking					0.0226*
Yes	1 (50.0)	19 (14.0)	145 (18.5)	430 (22.1)	
No	1 (50.0)	117 (86.0)	640 (81.5)	1,513 (77.9)	
Diabetes mellitus					0.0115*
Yes	0 (0.0)	10 (7.4)	113 (14.4)	331 (17.0)	
No	2 (100.0)	126 (92.6)	672 (85.6)	1,612 (83.0)	
Dialysis					0.6684
Yes	0 (0.0)	3 (2.2)	34 (4.3)	74 (3.8)	
No	2 (100.0)	133 (97.8)	751 (95.7)	1,869 (96.2)	

Characteristics^d^	PD (mm)				*P* value^c^
	< 4 (*n* = 152)	4 (*n* = 669)	5 (*n* = 817)	≥ 6 (*n* = 1,228)	
Age (years)					0.0002*
18–34	43 (28.7)	200 (30.3)	279 (35.0)	298 (24.7)	
35–64	77 (51.3)	307 (46.6)	355 (44.5)	630 (52.2)	
≥ 65	30 (20.0)	152 (23.1)	163 (20.5)	279 (23.1)	
Sex					0.0003*
Male	52 (34.2)	274 (41.0)	321 (39.5)	576 (47.2)	
Female	100 (65.8)	395 (59.0)	491 (60.5)	645 (52.8)	
Race					0.0383*
African American	16 (11.7)	119 (19.4)	150 (20.3)	247 (22.6)	
Asian	17 (12.4)	48 (7.9)	66 (9.0)	86 (7.9)	
Caucasian	48 (35.0)	237 (38.7)	246 (33.3)	348 (31.9)	
Hispanic	54 (39.4)	190 (31.1)	254 (34.4)	370 (33.9)	
Other	2 (1.5)	18 (2.9)	22 (3.0)	40 (3.7)	
Smoking					0.8934
Yes	29 (19.1)	142 (21.2)	174 (21.3)	250 (20.4)	
No	123 (80.9)	527 (78.8)	643 (78.7)	978 (79.6)	
Diabetes mellitus					0.7142
Yes	24 (15.8)	106 (15.8)	120 (14.7)	204 (16.6)	
No	128 (84.2)	563 (84.2)	697 (85.3)	1,024 (83.4)	
Dialysis					0.0134*
Yes	5 (3.3)	40 (6.0)	24 (2.9)	42 (3.4)	
No	147 (96.7)	629 (94.0)	793 (97.1)	1,186 (96.6)	

*P* values labeled in bold and with an asterisk (*) show statistical significance (*P* < 0.05).

^a^The analyses included only the 2866 subjects who had periodontal re-evaluation following non-surgical periodontal therapy.

^b^Missing values: 2 for age and 9 for race at CAL 1–2 mm; 19 for age, 2 for sex, and 78 for race at CAL 3–4 mm; 32 for age, 10 for sex, and 201 for race at CAL ≥ 5 mm.

^c^Chi-squared test was used to compare distributions of characteristics among categories of CAL and PD.

^d^Missing values: 2 for age and 15 for race at PD < 4 mm; 10 for age and 57 for race at PD 4 mm; 20 for age, 5 for sex, and 79 for race at PD 5 mm; 21 for age, 7 for sex, and 137 for race at PD ≥ 6 mm.

Multinomial logistic regression analysis was performed separately for CAL and PD to determine whether age, sex, race, smoking, self-report of DM, and dialysis were independently associated with CAL and PD categories at re-evaluation (Table 6). For regression analysis of CAL categories, the odds of CAL ≤ 2 mm and 3–4 mm were compared with CAL ≥ 5 mm as the reference category (Table 6). The age groups 35–64 years and ≥ 65 years had decreased odds of being in the CAL ≤ 2 mm and 3–4 mm categories compared with being in the CAL ≥ 5 mm category (*models 1 and 2*). Female sex had an increased odds of being in the CAL 3–4 mm category compared with being in the CAL ≥ 5 mm category (*model 2*). Non-smoking status increased the odds of being in the CAL ≤ 2 mm and 3–4 mm categories (*models 1 and 2*), and having other race decreased the odds of being in the CAL 3–4 mm category (*model 2*), compared with being in the CAL ≥ 5 mm category.

**Table 6:** Multinomial logistic regression of CAL and PD at re-evaluation^a^.

Characteristic	Model 1^b^ CAL ≤ 2 mm	Model 2^b^ CAL 3–4 mm	Model 3^c^ PD < 4 mm	Model 4^c^ PD 4 mm	Model 5^c^ PD 5 mm
	OR (95% CI)	OR (95% CI)	OR (95% CI)	OR (95% CI)	OR (95% CI)
Age (years)					
18–34	*Reference*				
35–64	**0.19 (0.13, 0.29)**	**0.37 (0.30, 0.46)**	0.83 (0.54, 1.28)	**0.71 (0.55, 0.90)**	**0.60 (0.48, 0.76)**
≥ 65	**0.13 (0.07, 0.25)**	**0.25 (0.18, 0.33)**	0.85 (0.49, 1.48)	0.79 (0.58, 1.07)	**0.63 (0.47, 0.84)**
Sex					
Male	*Reference*				
Female	1.35 (0.91, 2.00)	**1.51 (1.24, 1.83)**	**1.67 (1.14, 2.44)**	**1.32 (1.07, 1.63)**	**1.45 (1.19, 1.78)**
Race					
Caucasian	*Reference*				
Asian	0.53 (0.24, 1.21)	0.74 (0.52, 1.07)	1.44 (0.77, 2.68)	0.80 (0.53, 1.20)	1.04 (0.71, 1.52)
African American	1.22 (0.72, 2.08)	1.03 (0.79, 1.33)	**0.43 (0.24, 0.78)**	**0.66 (0.50, 0.88)**	0.80 (0.62, 1.05)
Hispanic	0.88 (0.55, 1.42)	0.81 (0.64, 1.02)	0.89 (0.57, 1.40)	**0.68 (0.52, 0.88)**	0.81 (0.63, 1.03)
Other	0.30 (0.07, 1.32)	**0.51 (0.28, 0.92)**	0.35 (0.08, 1.51)	0.64 (0.35, 1.16)	0.69 (0.39, 1.22)
Smoking					
Yes	*Reference*				
No	**1.96 (1.14, 3.37)**	**1.33 (1.04, 1.69)**	1.06 (0.65, 1.73)	0.97 (0.75, 1.25)	0.93 (0.73, 1.19)
Diabetes mellitus					
Yes	*Reference*				
No	1.69 (0.83, 3.44)	0.90 (0.69, 1.17)	1.06 (0.62, 1.79)	0.96 (0.73, 1.28)	0.92 (0.70, 1.20)
Dialysis					
Yes	*Reference*				
No	1.19 (0.36, 3.93)	0.74 (0.46, 1.17)	1.15 (0.40, 3.29)	0.57 (0.34, 0.91)	1.22 (0.70, 2.13)

OR (95% CI) values not crossing 1.0 are highlighted in bold.

^a^The analyses included only the 2866 subjects who had periodontal re-evaluation following non-surgical periodontal therapy.

^b^For analysis of CAL, the reference category was CAL ≥ 5 mm.

^c^For analysis of PD, the reference category was PD ≥ 6 mm.

For regression analysis of PD categories, the odds of PD < 4 mm, 4 mm, and 5 mm were compared with PD ≥ 6 mm as the reference category. The 35–64 years age group was associated with decreased odds of being in the PD 4 mm and 5 mm categories (*models 4 and 5*), and the ≥ 65 years age group was associated with decreased odds of being in the PD 5 mm category (*model 5*), compared with being in the PD ≥ 6 mm category. Female sex was associated with increased odds of being in the PD < 4 mm, 4 mm, and 5 mm categories compared with the PD ≥ 6 mm category (*models 3, 4, and 5*). Race was also associated with the PD category. African-American race was associated with decreased odds of being in the PD < 4 mm and 4 mm categories (*models 3 and 4*), and being Hispanic was associated with decreased odds of being in the PD 4 mm category (*model 4*), compared with being in the PD ≥ 6 mm category.

## Discussion

The present study aimed to address conflicting literature reports on the role of age and sex in the outcomes of NSPT. A study of 75 U.S. adult patients (aged ≥ 30 years) showed that 6 months after NSPT, twice as many all-age males had “active” probing sites that displayed periodontitis progression (evidenced by ≥ 2.5 mm CAL at ≥ 2 probing sites) compared with all-age females (29 vs. 15%, respectively). The same study also showed that twice as many older patients (60–69 years) had “active” probing sites compared with patients aged 50–59 years (56 vs. 27%, respectively, risk ratio 2.8) independent of baseline periodontal determinants^16^. Another study of 98 British patients with periodontitis (mean age 53 years) and undergoing NSPT, surgical periodontal therapy (when indicated), and periodontal maintenance for 5–10 years showed that the confounder-adjusted tooth loss, a terminal outcome of periodontitis, was significantly associated with older age (OR 1.11; 95% CI 1.06, 1.16; *P* < 0.001), whereas the male sex had a trend for greater odds of tooth loss (OR 2.00; 95% CI 0.95, 4.18; *P* = 0.068)^17^. Conversely, a retrospective study of 117 Italian patients with slowly progressing (former “chronic”) and rapidly progressing molar-incisor (former “aggressive”) phenotypes of periodontitis who received at least 3 sessions of NSPT showed no significant differences in treatment outcomes between younger and older subjects (mean age 35 and 59 years, respectively)^18^. Similarly, a study of 172 Swiss patients undergoing periodontal maintenance for 3–27 years revealed no significant differences in the progression of periodontal breakdown (OR 1.2; 95% CI 0.6, 2.6; *P* = 0.571) and tooth loss (OR 1.1; 95% CI 0.7, 1.9; *P* = 0.593) between males and females^19^. A systematic review with a meta-analysis of other studies showed that in patients who underwent NSPT, surgical periodontal therapy (when indicated), and periodontal maintenance for ≥ 3 years, the odds of tooth loss were significantly associated with older age (OR 1.05; 95% CI 1.03, 1.08; *P* < 0.05) but not with male sex (OR 0.95; 95% CI 0.86, 1.05; *P* ≥ 0.05)^20^.

The present study demonstrated that, at baseline (prior to NSPT), severe periodontitis (evidenced by CAL ≥ 5 mm) was significantly associated with middle-age and older age (35–64 years and ≥ 65 years, respectively). Severe periodontitis (evidenced by PD ≥ 6 mm) was significantly associated with middle age (35–64 years). In general, these results are consistent with the 2009–14 NHANES-based studies that showed that severe periodontitis (defined as the presence of ≥ 2 interproximal sites with ≥ 6 mm CAL) was more prevalent in middle-aged individuals (30–64 years) compared to older (≥ 65 years) adults (14.5 vs. 9.0%, respectively)^11^. Findings at re-evaluation were similar to those at baseline suggesting similar mechanisms underlying age-associated differences in severe periodontitis at baseline and re-evaluation. Since periodontitis develops and progresses as a result of the improper balance between periodontal pathogens and the host immune system, it is essential to understand whether changes to periodontal microbiota and/or immune responses in older vs. younger individuals would result in increased prevalence of periodontitis and clinically significant impact in the outcome of periodontal treatment. A recent cross-sectional study that included older untreated individuals (aged ≥ 65 years) showed that plaque score and bleeding on probing (surrogate markers of oral hygiene conditions and gingival inflammation, respectively) increased with age^21^. A large-scale study showed no significant differences in the composition of subgingival microbiota of younger and older individuals with periodontitis^22^, whereas other studies showed that older adults had a higher prevalence of *P. gingivalis* and *P. intermedia*^23,24^. These results may suggest that the increased periodontal breakdown could be due to systemic host factors rather than oral microbiota differences. Older adults often present with age-associated impaired adaptive immune system responses (senescence) and persistent low-grade inflammation (inflamm-aging) resulting from various cellular and humoral mechanisms (“age-related susceptibility” hypothesis)^25–30^. Indirectly, this is in agreement with older literature that suggested that the increased susceptibility of older patients to periodontitis was more critical for the rate of destruction than the duration of time the periodontal tissue was exposed to the dental biofilm^31^. Another recent study showed that older adults who maintain healthy lifestyles had significantly lower tooth-specific periodontal disease incidence/progression compared to those who do not maintain a healthy lifestyle (OR 0.61; 95% CI 0.39, 0.95)^14^.

The present study also demonstrated that males had a significantly higher prevalence of severe periodontitis (evidenced by CAL ≥ 5 mm) at baseline (prior to NSPT) and re-evaluation (higher odds of being in the CAL ≥ 5 mm or PD ≥ 6 mm category). Similarly, the 2009–14 NHANES-based studies showed that severe periodontitis (defined as the presence of ≥ 2 interproximal sites with ≥ 6 mm CAL) was significantly higher in all-age males compared to all-age females (11.5 vs. 4.3%, respectively)^11^. A retrospective study that included 858 Swedish subjects aged ≥ 60 years showed that periodontitis (defined as ≥ 5 mm interproximal CAL in ≥ 30% probing sites) was more prevalent in males (OR 1.8; CI 1.3, 2.4)^32^. Several mechanisms were proposed to underlie sex-related differences in the prevalence and/or severity of periodontitis. For example, similar to older adults, poorer oral hygiene in males could play an essential role in such differences. A large-scale 2017–18 NHANES-based study that included 4,741 adult participants (mean age 53.7 years) showed that males exercised worse home oral hygiene care (flossed significantly less frequently) and had less frequent professional dental care visits compared to females^33^, and both these behavioral factors were significantly associated with periodontitis^11^. In addition, sex-specific innate immune responses to microbial pathogens, possibly due to differential expression of toll-like receptors, X chromosome-linked innate immune genes, and microRNAs were reported, further suggesting that interactions of periodontal pathogens with the host immune system can be different in males and females^34^. Sex-associated differences in the association between periodontitis and type 2 DM^35^, metabolic syndrome^36^, myocardial infarction^37^, ischemic heart disease^32^, and mortality^32^, were also reported.

Contrary to the observations at baseline that males were more likely to be in the ≥ 5 mm CAL category than other CAL categories, the magnitude of the changes (Δ) in CAL and PD between baseline and re-evaluation was similar between males and females. However, logistic regression showed that males had significantly higher odds of being in the CAL ≥ 5 mm and PD ≥ 6 mm categories at re-evaluation compared to females. Mechanisms underlying differences in the prevalence and/or severity of periodontitis between males and females at re-evaluation may be similar to those at baseline. Since these differences might be primarily related to differences in oral hygiene conditions, plaque scores at baseline and re-evaluation would be an important clinical determinant to include in our future studies.

The average change in CAL and PD between baseline and re-evaluation was less than 1 mm for all strata, based on the demographic characteristics analyzed. Despite the small magnitude of the mean change, age, race, and smoking had statistically significant associations with the size of the mean change in linear regression analysis. Elderly (≥ 65 years) patients had a smaller mean change in PD than younger (18–34 years) patients; non-smokers had a smaller mean change in CAL than smokers; African Americans had a greater mean change in CAL and PD than Caucasians; Asians had a greater mean change in CAL than Caucasians. Statistical versus clinical significance is an important matter when deciding on the importance of outcomes of periodontal therapy, and approaches to evaluate periodontal status and considerations in determining clinical significance have been extensively described^38,39^. Statistical analysis can be affected by various factors, including statistical rarity, magnitude of an observed effect, characteristics of the study population, and incongruity with the response to therapy^39^. While the results of the present study indicate statistical significance and show good evidence of a true effect, they can be of limited importance due to the relatively small magnitude of the changes. This small magnitude could be affected by various factors that include our study limitations.

Although it was not the focus of the present study, significant associations were found between severe periodontitis (PD ≥ 6 mm) and the African American race (but not other races) at baseline. A higher prevalence of severe periodontal breakdown in African Americans was reported in large-scale epidemiological studies^11,40,41^. Similar to the baseline, there was a significantly higher proportion of African Americans in the severe periodontitis category at re-evaluation. In addition, linear regression analysis showed that African American race was associated with slight but statistically significant differences in CAL and PD values at re-evaluation compared to baseline. Similar to sex, differences in plaque scores by race or socioeconomic status at baseline and re-evaluation could provide further insight into race-associated responses to NSPT.

The strengths of the study include a large number of participants (*n* = 2866) treated over a multi-year (13 years) period at this academic center, full-mouth periodontal charts completed at baseline and re-evaluation, and the use of definitions and cutoffs of periodontitis consistent with the current classification of periodontal diseases. Limitations include a single center database, the retrospective study design and its associated inherited limitations (e.g., the lack of the investigator’s control over the collected data), missing data on potential confounders (such as gingival bleeding and plaque scores, and socio-economic status), the self-reported status of reported systemic diseases, and the short-term post-NSPT evaluation time point. In addition, characteristics of the study population indicate that the participants had high mean tooth loss at baseline and consequently represent a patient cohort with more severe periodontal breakdown. The high tooth loss at baseline also suggests that patients referred to an academic center setting likely represent a cohort with a greater proportion of severe periodontitis, and the interpretation of the study’s results should be considered in that context. NSPT providers at this academic center included a mix of dental students, postdoctoral trainees, and periodontal specialists, and so the variation in the level of expertise and experience of the providers could have impacted NSPT effectiveness and study outcomes. Because this was a retrospective study that did not calibrate provider experience or stratify analyses by the level of experience of the providers, no comment can be made regarding NSPT outcomes according to level of provider experience. Providers at academic dental centers usually have varied levels of experience; therefore, our observations are likely to reflect results that can be achieved in the setting of an academic center. Finally, since only patients with the most recent treatment episodes were included in the study, some patients might have several episodes of NSPT due to a poorer response to the therapy, which could impact the reported findings. To partially address these limitations, a multi-center study that includes dental databases from other U.S. dental schools should be performed and is currently in progress. To address these limitations further, a prospective randomized controlled study that includes non-surgical and surgical treatment modalities with accurately documented data at each treatment phase should be conducted.

## Conclusions

Despite the minor changes in CAL and PD between baseline and re-evaluation, the magnitude of these changes was significantly associated with age and other demographic factors. At both baseline and re-evaluation, older age and male sex were associated with higher CAL values, and older age was associated with higher PD values, indicating that the correlation of age and sex with CAL and PD categories was not changed by NSPT. Additionally, at re-evaluation, African American race was associated with higher PD, and smoking was associated with higher CAL, suggesting that these factors also have an impact on the outcomes of NSPT. Personalized strategies should be developed to achieve successful outcomes of NSPT in these vulnerable populations.

## Data Availability

The raw datasets used and/or analyzed during the current study are available from the corresponding author upon reasonable request and subject to clearance by the IRB of the University of Texas Health Science Center (Houston, TX, USA).
